# Side-effects of COVID-19 vaccines among the Saudi population: A cross-sectional study

**DOI:** 10.15537/smj.2022.43.4.20210905

**Published:** 2022-04

**Authors:** Ebtehaj S. Almughais, Ali H. Alharbi, Hadi A. Aldarwish, Areeb F. Alshammari, Razan S. Alsuhaymi, Jumanah A. Almuaili, Atheer M. Alanizy

**Affiliations:** *From the Department of Family & Community Medicine (Almughais), College of Medicine and from the College of Medicine (Alharbi, Aldarwish, Alshammari, Alsuhaymi, Almuaili, Alanizy), University of Hail, Hail, Kingdom of Saudi Arabia.*

**Keywords:** COVID-19, side-effects, Pfizer, AstraZeneca, Saudi Arabia

## Abstract

**Objectives:**

To measure and assess the side-effects of Pfizer/BioNTech and AstraZeneca vaccines on residents of Saudi Arabia, as well as provide a database that gives insight into the relative safety of these 2 COVID-19 vaccines.

**Methods:**

A community-based cross-sectional study was conducted to determine the side-effects of the two COVID-19 vaccines. The study was initiated on the 5th of June 2021 at Hail University, Hail, Saudi Arabia. The information was collected through an online survey designed on Google forms. The questionnaire was pre-tested for validity, with all information carefully reviewed.

**Results:**

The study included 2,530 participants from different regions of Saudi Arabia, with a mean age of 26.9 ± 12.4 years old. The most common vaccine among the study group was Pfizer, which 73.8% of the population were provided; the remaining 26.2% received the AstraZeneca vaccine. Regarding the Pfizer vaccine, the common systemic side-effects followed the first dose, included headaches, followed by muscle pain, fever, and joint pain. Those who had the AstraZeneca vaccine reported a few more side-effects. For example, during the first dose fever was reported as the most common side-effect, followed by headache, muscle pain and fatigue.

**Conclusion:**

The present study confirmed that vaccine side-effects are more frequently reported by smokers and those who received the AstraZeneca vaccine. Further studies are needed to acquire a better understanding of the association between risk factors and the experiencing of post-vaccine side-effects.

**S**evere Acute Respiratory Syndrome Coronavirus 2 (SARS-CoV-2) is a positive-sense single-stranded RNA virus (+ssRNA) which is the cause of the current Coronavirus Disease 2019 (COVID-19) pandemic. SARS-CoV-2 was first identified in Wuhan City, China, in late 2019. Thereafter, it quickly spread around the planet, with ~14 million active cases and ~582,000 deaths recorded as of July 2020.^1^ Therefore, there has been an urgent international demand for the scientific community to develop an effective vaccine.

In September 2020, the World Health Organization declared the launch of several COVID-19 vaccines.^2^ On December 31, 2020, the mRNA vaccine produced by Pfizer, and on February 15, 2021, the ChAdOx1 nCoV19 vaccine produced by AstraZeneca Oxford, were approved for emergency use.^3^ Research found that the AstraZeneca was 70% effective and Pfizer vaccine was 95% effective.^4,5^

Soon after the vaccines were developed, Saudi Arabia took the initiative and promptly provided these 2 vaccines to the public over 3 phases. Phase one was targeted towards individuals over 65 years of age and front-line health care workers (HCWs). Phase 2 targeted individuals over 50 years of age and other health care practitioners. Last, phase 3 targeted all citizens and residents in Saudi Arabia.^6^

A major obstacle in managing the COVID-19 pandemic is vaccination hesitancy (example, unwillingness to get vaccinated). Previous studies in Qatar found that 20% and Kuwait 26.2% expressed vaccine hesitancy.^7,8^ Researchers see this as a significant public health challenge, which is fueled by misleading and inaccurate information on vaccine safety and efficacy.^9^

In general, many concerns, questions, and arguments were raised regarding the COVID-19 vaccine program by the general population of Saudi Arabia, regarding how safe the approved vaccines were. There is however limited data and literature concerning each vaccine’s side-effects, along with the influence of demographic factors such as age, gender, smoking, and comorbidities. Therefore, the objective of the present study was to investigate the safety and adverse effects of the Pfizer and Oxford-AstraZeneca vaccines among Saudis who had received one of them.

## Methods

A community-based cross-sectional study was approved by the Research Ethics Committee, University of Hail, Hail, Saudi Arabia (No H-2021-177, dated 20/9/2021). The study was carried out to determine the vaccine side-effects from June to September 2021. The information was collected through an online survey designed on Google forms, which was written in Arabic and distributed to participants via 2 social media platforms (namely, WhatsApp and Twitter). Participation was voluntary and anonymous. Participants’ information was kept confidential according to Google’s privacy policy. The first page of the survey included a description of study, along with a statement regarding informed consent. The principles of the Declaration of Helsinki were followed to insure the rights of the human participants.

By using the formula ss=(Z2×p×q)/c2 the optimal sample size for conducting this study was determined to be 384 participants from each province (central, northern, southern, eastern, western). Where ss=sample size, Z=1.96, *p*=0.5, q=(1-p) =0.5, c=sampling error of 5%. In total, 2,530 respondents participated. Inclusion criteria included participants aged 18 years or over, who had received either the Pfizer or Oxford-AstraZeneca vaccine and willing to participants in the study. Respondent below the age of 18 and incomplete submissions were excluded.

### Development and application of the questionnaire

Questionnaires were developed after undertaking a literature review. Many post-vaccination side-effects were identified and covered in this study. Information was collected regarding participants’ demographic data, including their age, gender, height, and weight. Furthermore, assessments were made of their past medical history and general health status prior to vaccination. The prevalence of infection rates among the vaccinated population and their intention to take the second dose was also recorded. The second section of the study then considered the side-effects associated with the 2 COVID-19 vaccines under investigation. These were divided into general side-effects such as headaches, fatigue, and fever, along with local side-effects such as pain, tenderness, and swelling. Participants’ consent was secured before they completed the questionnaire.

### Statistical analysis

Data was collected, reviewed and then inputted into the Statistical Package for Social Sciences for Windows, version 21 (IBM Corp., Armonk, NY, USA). All statistical methods used were two tailed, with an alpha level of 0.05 considering significance if the *p*-value was <0.05. Descriptive analysis was performed by prescribing frequency distribution and percentages for study variables, including respondents’ personal data, history of COVID-19 infection, vaccination data, and post-vaccination side-effects. Cross tabulation showing the distribution of participants’ post-vaccination side-effects by their bio-demographic data, medical data, history of COID-19 infection, and vaccine type, was conducted via a Pearson Chi-square test for significance and exact probability due to the small frequency distribution.

## Results

The study included 2,530 participants from different regions of Saudi Arabia, 751 from the Western region, 508 from the Northern region, 467 from the Eastern region, 427 from the Southern region, and 377 from the Central region, with a mean age of 26.9±12.4 years old (Table 1).

**Table 1:** - Bio-demographic data of study’s vaccinated population.

Bio-demographic data	n	(%)
***Age in years***		
18-25	1212	(47.9)
26-35	703	(27.8)
36-50	497	(19.6)
51-60	87	(3.4)
>60	31	(1.2)
Gender		
Male	1037	(41.0)
Female	1493	(59.0)
***Educational level***		
Primary or less	26	(1.0)
Intermediate / secondary	567	(22.4)
University	1937	(76.6)
***Work***		
Healthcare workers	677	(26.8)
Others	1853	(73.2)
Body mass index		
Underweight	170	(6.7)
Normal	1224	(48.4)
Overweight	680	(26.9)
Obese	279	(11.0)
Morbid obesity	177	(7.0)
***Smoking***		
Yes	412	(16.3)
No	2118	(83.7)
***Chronic health problems***		
Yes	372	(14.7)
No	2158	(85.3)
***Had any type of allergy***		
Yes	366	(14.5)
No	2164	(85.5)

Regarding the participants’ COVID-19 infection history, approximately 19% of the study participants had previously had a positive test for COVID-19. The most taken vaccine among the study group was the Pfizer vaccine, which 73.8% of the participants received. Among those who had received a single dose, 62.3% agreed to have the second dose. 94.1% of the vaccinated respondents had no COVID-19 infection after the vaccine. Most participants (87.5%, 2213) had at least one of the reported post-vaccination side-effects (Table 2).

**Table 2:** - COVID-19 history of infection and vaccination data among vaccinated population.

COVID-19 infection and vaccination data	n	(%)
***Previously had positive test for COVID-19***		
Yes	488	(19.3)
No	2042	(80.7)
Type of vaccine		
Pfizer	1868	(73.8)
AstraZeneca	662	(26.2)
***Agree to have second dose of COVID-19 vaccine***		
Yes	1575	(62.3)
No	435	(17.2)
Had both doses	520	(20.6)
***Infected with COVID-19 after vaccination***		
After 1st dose	132	(5.2)
After 2nd dose	18	(0.7)
No	2380	(94.1)
***Post-vaccination side-effects***		
Yes	2213	(87.5)
No	317	(12.5)

A wide spectrum of post vaccination side-effects among the Saudi population were studied. Table 3 reveals the frequency of these side-effects. Regarding the Pfizer vaccine, the most reported systemic side-effects came after the first dose. These were headache (34.7%) followed by muscle pain (31.7%). The least reported systemic side-effects were nausea (10.2%) and diarrhea (6.7%). Considering localized side-effects, the most reported were local injection pain with touch (70%). Axillary lymphadenopathy was only reported among 4.2% of the participants. Compared with the Pfizer vaccine, those who had the AstraZeneca vaccine, 62.5% of participants complained of fever following the first dose, followed by headache (55.9%). The least reported side-effect was nausea (21.5%). Regarding the local side-effects, 68.3% of the participants complained of local injection pain. The least reported local side-effect was axillary lymphadenopathy 5%.

**Table 3:** - Distribution of post vaccination side effects among study population by vaccine type, Saudi Arabia.

Type of vaccine	Side effects	None		After 1st dose		After 2nd dose		After both doses	
		n	(%)	n	(%)	n	(%)	n	(%)
Pfizer	***Systemic side effects***								
	Headache	1108	(59.3)	648	(34.7)	60	(3.2)	52	(2.8)
	Fatigue	1433	(76.7)	352	(18.8)	57	(3.1)	26	(1.4)
	Fever	1367	(73.2)	360	(19.3)	106	(5.7)	35	(1.9)
	Chills and tremors	1635	(87.5)	177	(9.5)	41	(2.2)	15	(0.8)
	Joint pain	1456	(77.9)	326	(17.5)	51	(2.7)	35	(1.9)
	Muscle pain	1142	(61.1)	592	(31.7)	70	(3.7)	64	(3.4)
	Diarrhea	1742	(93.3)	103	(5.5)	21	(1.1)	2	(0.1)
	Nausea	1676	(89.7)	154	(8.2)	27	(1.4)	11	(0.6)
	***Local SE***								
	Local pain	888	(47.5)	826	(44.2)	45	(2.4)	109	(5.8)
	Local edema	1428	(76.4)	365	(19.5)	32	(1.7)	43	(2.3)
	Local pain with touch	560	(30.0)	1091	(58.4)	56	(3.0)	161	(8.6)
	Itching	1652	(88.4)	185	(9.9)	20	(1.1)	11	(0.6)
	Axiliary lymphadenopathy	1790	(95.8)	56	(3.0)	18	(1.0)	4	(0.2)
	Local redness	1561	(83.6)	255	(13.7)	27	(1.4)	25	(1.3)
	Local bruising	1714	(91.8)	128	(6.9)	16	(0.9)	10	(0.5)
	Local hotness	1378	(73.8)	409	(21.9)	31	(1.7)	50	(2.7)
AstraZeneca	***Systemic side effects***								
	Headache	272	(41.1)	370	(55.9)	8	(1.2)	12	(1.8)
	Fatigue	367	(55.4)	267	(40.3)	9	(1.4)	19	(2.9)
	Fever	218	(32.9)	414	(62.5)	7	(1.1)	23	(3.5)
	Chills and tremors	386	(58.3)	257	(38.8)	6	(0.9)	13	(2.0)
	Joint pain	394	(59.5)	246	(37.2)	11	(1.7)	11	(1.7)
	Muscle pain	312	(47.1)	328	(49.5)	10	(1.5)	12	(1.8)
	Diarrhea	582	(87.9)	70	(10.6)	7	(1.1)	3	(0.5)
	Nausea	513	(77.5)	142	(21.5)	3	(0.5)	4	(0.6)
	***Local SE***								
	Local pain	284	(42.9)	343	(51.8)	5	(0.8)	30	(4.5)
	Local edema	491	(74.2)	158	(23.9)	6	(0.9)	7	(1.1)
	Local pain with touch	166	(25.1)	452	(68.3)	8	(1.2)	36	(5.4)
	Itching	568	(85.8)	87	(13.1)	4	(0.6)	3	(0.5)
	Axillary lymphadenopathy	629	(95.0)	28	(4.2)	3	(0.5)	2	(0.3)
	Local redness	551	(83.2)	100	(15.1)	4	(0.6)	7	(1.1)
	Local bruising	585	(88.4)	67	(10.1)	5	(0.8)	5	(0.8)
	Local hotness	466	(70.4)	179	(27.0)	3	(0.5)	14	(2.1)

Table 4 highlights the determinants of developing post-vaccination side-effects among the studied Saudis population. Approximately 88.5% of participants aged 51 years or older had side-effects after vaccination, compared with 83.1% of those aged 35 to 50 years old, with a recorded statistical significance of *p*=0.017. Approximately 93.2% of smokers had post-vaccine side-effects compared with 86.4% of non-smokers (*p*=0.001). Side-effects after vaccination were also reported by 88.7% of persons with no history of COVID-19 infection, in comparison with 82.2% among those who had a history of COVID-19 infection (*p*=0.001). In addition, 94.3% of participants who had the AstraZeneca vaccine reported post-vaccination side-effects, in comparison with 85.1% of those who had the Pfizer vaccine (*p*=0.001).

**Table 4:** - Determinants of developing post-vaccination side-effects among study participants.

Factors	Yes		No		P-value
	n	(%)	n	(%)	
***Age in years***					
18-25	1081	(89.2)	131	(10.8)	0.017*
26-35	615	(87.5)	88	(12.5)	
36-50	413	(83.1)	84	(16.9)	
51-60	77	(88.5)	10	(11.5)	
>60	27	(87.1)	4	(12.9)	
***Gender***					
Male	897	(86.5)	140	(13.5)	0.219
Female	1316	(88.1)	177	(11.9)	
***Nationality***					
Saudi	2097	(87.2)	309	(12.8)	0.036*
Non-Saudi	116	(93.5)	8	(6.5)	
***Body mass index***					
Non obese	1211	(86.9)	183	(13.1)	0.314
Overweight / obese	1002	(88.2)	134	(11.8)	
***Educational level***					
Below university	456	(76.9)	137	(23.1)	0.001*
University	1757	(90.7)	180	(9.3)	
***Work***					
HCWs	606	(89.5)	71	(10.5)	.061
Others	1607	(86.7)	246	(13.3)	
***Smoking***					
Yes	384	(93.2)	28	(6.8)	0.001*
No	1829	(86.4)	289	(13.6)	
***Chronic health problems***					
Yes	332	(89.2)	40	(10.8)	0.262
No	1881	(87.2)	277	(12.8)	
***Had any type of allergy***					
Yes	325	(88.8)	41	(11.2)	0.407
No	1888	(87.2)	276	(12.8)	
***Previously had positive test for COVID-19***					
Yes	401	(82.2)	87	(17.8)	0.001*
No	1812	(88.7)	230	(11.3)	
***Type of vaccine***					
Pfizer	1589	(85.1)	279	(14.9)	0.001*
AstraZeneca	624	(94.3)	38	(5.7)	
***Infected with covid-19 after vaccination***					
After 1st dose	121	(91.7)	11	(8.3)	0.288^#^
After 2nd dose	15	(83.3)	3	(16.7)	
No	2077	(87.3)	303	(12.7)	

P: Pearson X^2^ test, #: exact probability test, *p<0.05 (significant)

The factors mentioned in Table 5 were the most significant predictors for post-vaccination side-effects. Those who were female, overweight, non-Saudi, university educated, smokers, who have a chronic disease or took the AstraZeneca vaccine had more chance of developing post-vaccine side-effects (OR>1). On the other hand, those who were older or who previously had a positive test for COVID-19 had less risk than the others (OR<1).

**Table 5:** - Multiple stepwise logistic regression for developing post-vaccination side-effects among study participants.

Factor	*P*-value	OR _A_	95% CI for OR	
			Lower	Upper
Female	0.004*	1.50	1.10	1.90
Old age	0.007*	0.80	0.70	0.90
Non-Saudi	0.048*	2.00	1.00	4.30
University education	0.001*	2.90	2.30	3.80
Smokers	0.001*	2.60	1.70	3.90
Have chronic health problems	0.027*	1.60	1.10	2.30
Previously had positive test for COVID-19	0.001*	0.60	0.40	0.80
AstraZeneca vaccine	0.001*	2.80	1.90	4.00

OR_A_: adjusted odds ratio, CI: confidence interval, *p<0.05 (significant)

## Discussion

Since vaccine production began, people have expressed concerns on the dangers and risks of administering them. This study was therefore carried out to assess the side-effects of the Pfizer and AstraZeneca vaccines among vaccinated Saudi populations. Out of 2530 participants, 2213 (87.5%) had at least one of the reported side-effects, while 317 (12.5%) reported no side-effects. The most common systemic side-effects found in this study were headache, muscle pain, fever, fatigue, and joint pain. These results are similar to other studies conducted by Riad et al^10^ and Zhu et al.^11^ Local injection pain was the most reported local side-effect. Similar data was also reported in a recent study.^12-14^ Data analysis identified several adjusted determinants for developing side-effects. These were: younger age, female, smokers, comorbidity, history of COVID-19 infection, and receiving the AstraZeneca vaccine.

The survey was distributed online. This can result in sampling biases regarding age, as older people are less likely to have internet access or be computer literate. Moreover, other studies have also found that older people (>55 years) were less likely to develop side-effects.^15-18^ This finding could be interpreted in terms of the immune system response. Immune systems are more efficient and stronger in younger people. Since the immune system can produce cytokines post-vaccination, which could have an inflammatory effect on blood vessels, muscles, and other tissues, this may therefore explain the prevalence of the development of side-effects in younger people more than in the elderly.^16^ However, in contrast to the assumption that the older you are, the less likely you are to developed side-effects, El Shitany et al^19^ found that Saudi people aged 60 and over had a significantly higher frequency of developing local side-effects, particularly pain in the injection site area (80.8% versus 68.6%: significant=0.0056). Furthermore, 50% of female participants were more prone to develop post-vaccine side-effects compared with males (adjusted odds ratio [AOR] 1.5,95%confidence interval [CI]: 1.1-1.9, *p*=0.004). This finding was also observed by Menni et al,^20^ for both Pfizer and AstraZeneca vaccines. Moreover, many other studies also observed this association.^19,21^ This is likely because COVID-19 vaccines work by stimulating the immune system, which can have more pronounced effects on females due to gender-based differences in immune response, as seen in vaccines such as bacille Calmette-Guerin, measles, mumps, and rubella, and Yellow fever vaccine along with many others.^22,23^

Watanabe et al^24^ measured antibody titers in smokers who received the Pfizer vaccine and found serum antibody titer concentrations were significantly lower when compared with expected values. In this study, smoking displayed a significant relation with developing more post-vaccine side-effects. 16.3% of study participants were smokers; 93.2% of them developed side-effects. Smokers have lower antibody titer concentrations, which can explain the increase of post-vaccine side-effects in this group. Moreover, chronic health problems also showed a positive association with developing side-effects (AOR 1.6,95%CI: 1.1-2.3, *p*=0.027). Furthermore, most other studies have also identified comorbidities as a significant factor.^25^ This can be due to the complicated and multifactorial nature of chronic diseases. For instance, in one study, obesity and hypertension were associated with lower antibody titer concentration 3 weeks after receiving the Pfizer vaccine.^24^ On the other hand, no significant association between high BMI and post-vaccine side-effects was found in this study.

The present study showed that people with COVID-19 history had lower odds of adverse effects after COVID-19 vaccination. On the other hand, most of the previous studies showed that prior COVID-19 infection had been associated with increase the risk of vaccination side effects. For instance, population studies in Iraq and the United Kingdom found that individuals with evidence of past SARS-CoV-2 infection were also more likely to have adverse effects to both vaccines than those without evidence of past infection.^20,26^ Although there is no clear explanation, previous research examined the antibody responses in 109 people. A total of 68 patients had never had COVID-19, whereas 41 had previously tested positive. The research indicated that people who had a history of COVID-19 infection had higher antibody concentrations than those who had never been infected.^27-29^

Concerning the type of vaccine, the AstraZeneca vaccine had more frequently reported side-effects than the Pfizer vaccine. Other studies have also found this.^30-33^ This finding is consistent with the claim that the mRNA vaccine has fewer side-effects than other types of vaccine. According to the clinical trials, there were no serious systemic side-effects after administration of mRNA vaccine, only headache and fatigue. Fever was noticed after the second dose in less than 16% of participants, which supports the view that mRNA vaccines are safe and have fewer side-effects.^34^ When comparing the first dose and the second dose of the vaccine, Hatem et al^32^ also found that side-effects are usually more pronounced after the first dose, as also highlighted by Riad et al.^28^ However, according to the Centers for Disease Control and Prevention (CDC), as well as other studies, side-effects can be more intense after the second dose.^35,5,19^ The CDC report that people with a history of allergic reactions should be vaccinated with great caution. Furthermore, people with a history of severe allergic reactions, such as anaphylaxis, should not receive the vaccine at this stage.^36^ However, in this study, it was found that a history of an allergic reaction was not a significant factor in developing side-effects. This study also found that heatlhcare workers (HCWs) were not more likely to develop side-effects. On the other hand, another recent study among HCWs found a wide range of post-vaccination symptoms, most of which were not life-threatening.^12^

### Study limitations

The study was reliant on self-reports by participants, snowballing sampling method was utilized which can lead to a biased study population. The main limitation of this study is that during the data collection period, the Saudi government only allowed persons who were over 60 years old, HCWs, and those with a few selected medical conditions, to take the second dose of the vaccine, However, now every age group can take the second dose. Unfortunately, this cannot be reflected in our study and the second dose side-effects cannot therefore be sufficiently represented. Furthermore, a randomized control study could be better at detecting any significant relationships between risk factors and developing post-vaccinated side-effects.

In conclusion, this study despite the high prevalence of side-effects after vaccination among participants, this study concluded that most post-vaccination side-effects are typical symptoms which are also found with other vaccines. The most common side-effects in both vaccines were headache, muscle pain, injection site pain, and local pain with touch, with these side-effects reported especially after the first dose. More frequent side-effects were reported by smokers and those who received the AstraZeneca vaccine.

This study therefore provides a database to inform people on the possibility of developing side-effects based on their gender, age, and the type of vaccine which is administered. However, further studies should be conducted to arrive at a better understanding of the association between risk factors and developing side-effects.
